# Can an Instrument Validated to Assess Parent–Child Interactions in the Laboratory Setting Be Applied to Home-Based Observations?

**DOI:** 10.3389/fped.2020.550922

**Published:** 2021-01-15

**Authors:** Helen H. Lee, Nadia Ochoa, Nia Moragne-O'Neal, Genesis F. Rosales, Oksana Pugach, Anuoluwapo Shadamoro, Molly A. Martin

**Affiliations:** ^1^Department of Anesthesiology, College of Medicine, University of Illinois at Chicago, Chicago, IL, United States; ^2^Institute for Health Research and Policy, University of Illinois at Chicago, Chicago, IL, United States; ^3^Division of Epidemiology and Biostatistics, School of Public Health, University of Illinois at Chicago, Chicago, IL, United States; ^4^University of Illinois at Chicago, Chicago, IL, United States; ^5^Department of Pediatrics, College of Medicine, University of Illinois at Chicago, Chicago, IL, United States

**Keywords:** mother-child interaction, toothbrushing, behavior, oral - general health, urban health

## Abstract

**Background:** The Toothbrushing Observations Scale (TBOS) was developed in a laboratory setting to measure child and parent behaviors during toothbrushing. However, we required an instrument to assess home based behaviors. We assessed the feasibility of applying TBOS to observations of parents and their child (<3 years of age) in urban homes.

**Methods:** Sample consisted of 36 families recruited from university and community pediatric dental/medical clinics and a Women, Infants, and Children center in Chicago as part of a pilot study for a larger clinical trial. The average age of children in our sample was 20.7 months. Most of the parent participants were mothers (90%), and 75% of the parents identified as Hispanic. Parent–child dyads were video-recorded during home-based toothbrushing activities and footage was reviewed by two independent TBOS coders.

**Results:** The TBOS instrument consists of 12 parent and 18 child items. We were able to code five parent and ten child items.

**Conclusion:** The feasibility of applying the TBOS measure to our study population was somewhat limited by factors related to home-based observations and the young age of children in our study. Instruments need to be validated across natural settings, such as the home, to increase the quality and accuracy of human behavioral data.

## Introduction

Developing protective oral health behaviors is an important aspect of maintaining children's oral health. The presence of regular, parent-assisted toothbrushing predicts better pediatric oral health outcomes (1). However, only half of children at high-risk for caries have regular toothbrushing habits (2, 3). Barriers to developing a regular toothbrushing routine may be rooted in parent–child interactions (4). Parental supervision behavior (5) and constructive parenting styles appear to contribute to establishing regular toothbrushing (4, 6). The Toothbrushing Observation System (TBOS) was developed to better discriminate between parent and child behaviors that contribute or hinder improving children's oral health. Higher parent TBOS scores (greater degree of parental adaptive behaviors) were associated with longer parent toothbrushing times and lower child caries, while higher child TBOS scores were associated with lower odds of parents' perception that toothbrushing was difficult (6).

Children's oral health is a reflection of social determinants of health (7, 8). Interventions to change behaviors on the individual level may be limited by the inability to also address family-wide influences on a child's oral health. Coordinated Oral health Promotion (CO-OP) Chicago, a cluster-randomized trial, addresses familial influences by testing the impact of community health workers (CHWs) on oral health in high-risk children under 3 years of age (NCT03397589). In preparation for this large clinical trial, pilot work was conducted to determine the feasibility of collecting behavioral oral health data in the home setting (3, 9).

This exploratory study represents a portion of the above-described pilot study. In the absence of an existing validated instrument that aligned with the requirements of our study (children <3 years of age, home observations), we needed to determine the feasibility of applying the TBOS, which was developed in a laboratory setting in school-aged children, to our study protocol. We were also interested in exploring the relationship between modified TBOS parent and child scores and outcomes related to oral health behaviors.

## Methods and Materials

### Study Population and Setting

Forty-five home observations were conducted between November 2016 and May 2017. Details of recruitment and enrollment have been previously reported (3, 9). In summary, research assistants (RA's) identified potential study participants during a clinic visit. Observations were conducted after obtaining informed consent during a home visit. Participants were recruited from university and community pediatric dental clinics, a pediatric medical clinic, and a Special Supplemental Nutrition Program for Women, Infants, and Children (WIC) center in Chicago. Inclusion criteria included: (1) parent > 18 years; (2) child <3 years; (3) child had >1 tooth; (4) parent lived with child a minimum of 5 days per week; and (5) English or Spanish speaking parent. Exclusion criteria included caregiver unable to participate or complete study protocol; caregiver previously approached/screened; child does not have teeth fully erupted.

### Measures

#### TBOS

The TBOS was designed to identify parent and child refusal and adaptive behavior management strategies during toothbrushing interactions. Parent items that measure adaptive behaviors include “laughed or smiled during toothbrushing” and “one or more positive messages to motivate.” Child items include behaviors such as “sucked, bit, or chewed on toothbrush” and “yelled or screamed” during toothbrushing. The TBOS was developed for observational use with young children—a cohort lacking in dental research (6). Given our younger targeted study population, the TBOS was the best measure found whose utility could be tested for our study protocol. The full TBOS (6) consists of 12 parent items and 18 child items coded with values 1 and 0. Items can be omitted from coding if not applicable to a given observation. Final parent and child scores are calculated by dividing the sum of values by the number of coded items. Scores range from 0 (no adaptive behaviors coded) to 1 (all adaptive behaviors coded). Based on our study design, five parent items and ten child items were identified to apply to field observations (Supplementary Table 1).

#### Oral Health Behaviors

Child oral health outcomes included duration since last dental visit (≤12 months vs. no visit/visit >12 months) and brushing frequency (>twice/day vs. <twice/day).

### Procedures

At participants' homes, parents were asked questions about child oral health behaviors. RAs then asked parents to demonstrate their child's typical toothbrushing routine. Video recording of the routine began as the parent prepared the brushing equipment (e.g., toothbrush, toothpaste) and ended upon either brushing completion or termination of the demonstration due to child noncompliance. Video data were collected using a handheld video recorder (Canon VIXIA HF R700). Participants were compensated with a cash incentive ($25 or 40 depending on the pilot's phase) and given toothbrushes and an oral health information sheet at the visit's end.

Video footage was stored in a secure computer drive and reviewed on an office desktop using Windows Media Player. Spanish dialogues were translated and transcribed into English by a bilingual RA. The English translations were used in conjunction with the video footage when coding. Two independent coders, not involved in the home data collection process, used the TBOS to discern and evaluate child and parent behaviors during toothbrushing. The process of coding each item and calculating parent and child scores was performed according to Collett's protocol (6).

### Analyses

Descriptive statistics were performed to characterize study participants. To determine scoring consistency across coders, we calculated intraclass correlation coefficients (ICC) to measure interrater reliability. Total TBOS scores for Parent and Child items were converted to standardized z-scores to allow for associations with outcomes per change in TBOS score standard deviation. Associations with outcomes were tested for TBOS parent and child scales separately using *T*-test and ANOVA, as appropriate. Statistical significance was determined by *p*-values below 0.05. Analyses were performed using SAS version 9.4 (Cary, North Carolina, USA).

## Results

Participants reflected the demographics of the recruitment sites as well as the targeted population for enrollment in the larger clinical trial (Table 1). The mean age of children was 20.7 months. Parent participants were predominantly mothers (90%). Small proportions of parents identified as White (11%) or Black (16%) race and 75% of parents identified as Hispanic in ethnicity. While 45 home observations were completed, only 36 videos (80%) were coded due to language or not consenting to a videorecording (3). In order to determine consistency in scoring across coders, we calculated intraclass correlation coefficients (ICC) as a measure of interrater reliability. TBOS parent items (*n* = 5) had ICC of 0.82 and TBOS children items (*n* = 10) had a slightly lower ICC of 0.72.

**Table 1:** Demographics of children and caregivers.

**Child–caregiver dyads *N* = 36**	
**Child demographics**	
Age (months), mean (SD)	20.7 (6.0)
Female *n*, (%)	26 (72.2)
**Caregiver demographics**	
Age (years), mean (SD)	31.3 (6.5)
Female n, (%)	34 (94.4)
Race *n*, (%)	
Non-Hispanic White	2 (5.6)
Non-Hispanic Black	6 (16.7)
Other	28 (77.8)
Hispanic *n*, (%)	27 (75.0)

We identified five out of 12 parent items and 10 out of 18 child items to apply to field observations for the larger clinical trial (Supplementary Table 1). The physical limitations of videorecording parent–child interactions in urban homes were quickly identified, as RAs reported that bathrooms were too small to accommodate four people (parent, child, RA filming, and second RA). Layouts and orientations of parent–child–camera varied, which also hindered consistency across recordings. Because recordings focused on the child's mouth, oftentimes video recordings captured parent's backside, which occasionally obscured a portion of the child's face. Parent and child behaviors were at least partially obscured in >50% of video-recordings. Due to these limitations, visual-based parent and child items were not coded. Other feasibility issues related to children's developmental stage and study design differences between TBOS and COOP. Certain child items reflected study design features specific to TBOS development that did not align with our study design or population. The TBOS study protocol began with playtime, then transitioned to toothbrushing activities. Our video recordings began with an invitation for the parent–child dyad to demonstrate their regular toothbrushing activities and did not test transitioning from play behaviors. “Easily transitioned to toothbrushing” as a child item was therefore not coded. Children in this study were younger and less verbal than those observed in the creation of the TBOS. The overwhelming majority (92%) of children in the TBOS study were >24 months of age, while the majority (64%) of children in our study were <24 months of age. We did not capture any verbal toothbrushing descriptions or brushing related questions from children. Likewise, “bossy” child behaviors toward parents implied a level of verbal engagement or the ability to observe a child directing their parent physically, which was hindered by impaired visualization of both the parent and child in our recordings. We did not observe any negative verbal behaviors from parents (“yelled at child,” “one or more negative remarks,” “made threats”).

We explored possible relationships between our modified TBOS and oral health behaviors (dental visits and toothbrushing frequency). We did not observe a relationship between modified parent or child TBOS scores and preventive dental visits or tooth brushing frequency (Table 2).

**Table 2:** Association between parent and child behaviors during toothbrushing and oral health behaviors.

**Sample *N* (36)**	***n* (%)**	**Parent TBOS^Ψ^ z-score, mean (SD)**	***t*-test (pooled) difference**	**Child TBOS^Ψ^ z-score, mean (SD)**	***t*-test (pooled) difference**
**Toothbrushing twice daily**					
No	9 (25.0)	−0.43 (0.63)	−0.57, *p* = 0.14	−0.32 (0.97)	−0.43, *p* = 0.27
Yes	27 (75.0)	0.14 (1.07)		0.11 (1.00)	
**Dental visit within last 12 months**					
No	12 (33.3)	−0.29 (0.77)	0.45, *p* = 0.21	0.27 (0.88)	−0.40, *p* = 0.26
Yes	24 (66.7)	0.15 (1.08)		−0.13 (1.05)	

*The Toothbrushing Observation System (TBOS) is an instrument developed in the laboratory setting to assess parent-child interactions during toothbrushing*.

Ψ *Parent and Child TBOS scores were modified from original measure (6) to reflect feasibility in applying to video-recordings in the home setting*.

## Discussion

The objective of this study was to explore the applicability of a validated measure within our study protocol, which entailed a younger study population, a home-based rather than laboratory setting, and differences in protocol for observations. Application of the TBOS measure to capture parent–child interactions during toothbrushing in urban low-income households was hindered by logistical barriers to data collection within urban homes as well as limitations in observing very young children. This required us to abbreviate the scorable TBOS items, which may have influenced this measure's ability to detect associations between parent–child brushing behaviors and outcomes. While modifying the TBOS measure raises validity concerns, per Collett, individual items can be removed to better suit the observations' conditions (6). This is a reasonable caveat to apply to our study when considering that human behavior and conceptualized constructs are likely to differ in natural vs. laboratory settings.

Subsequently, our pragmatic goals were achieved. We found that TBOS items that relied on age-appropriate child behaviors were largely not applicable to our population of children <3 years of age, mostly related to a lack of verbal skills. We also found that our study protocol, which entailed two research assistants conducting observations in urban homes, limited the feasibility of coding TBOS items that relied on visualization of parent and child interactions. Despite feasibility issues, we were able to capture many nonverbal child behaviors and many verbal parent behaviors. This is the first time, to our knowledge, that the TBOS has been applied to characterize behaviors in the home with very young children.

The data also allowed us to examine the preliminary relationships in some of the variables of interest for the larger study. We did not observe an association between the degree of parent and child adaptive behaviors and frequency of toothbrushing or duration of last dental visit. The relationship between TBOS scores, oral health behaviors, as well as will be further tested in a full clinical trial, as well as variables to measure parental psychosocial factors.

Our study findings are susceptible to some limitations. First, associations between modified TBOS scores and outcomes were limited by the small study sample size. Statistical analysis should be interpreted with caution because of potential high variability and uncertainty of parameter estimates. Second, the potential for response and observation bias may account for some of our findings. Participants might have been uncomfortable or self-aware during video recordings, which could alter natural behaviors during toothbrushing. The absence of parents yelling or making verbal threats during toothbrushing, as was observed in the laboratory setting (6), might be due to participants' altering behavior in the presence of research assistants and a videorecorder (Hawthorne effect and/or response bias). While observed behaviors may have been biased by methods of collection (presence of strangers in the home, participant need to please research team), we cannot eliminate child behaviors as influencing factors in establishing regular and high-quality toothbrushing habits. Finally, our results are prone to selection bias as participants were based upon a convenience sample, which may reflect the uneven distribution of participant race/ethnicity and gender.

This study's findings point to the importance of testing instruments across settings to increase the quality of human behavioral data collected in research. This work furthers our understanding on how data collection processes may reflect an accurate representation of the constructs of interest in a given research study—the importance of which is critical to testing whether different interventions work. When advocating for community, home-based interventions for novel groups, it makes the need for accurate data collections tools urgent to properly evaluate such interventions' efficacy.

## Data Availability Statement

The datasets presented in this article are not readily available because the data are currently still being used by the study investigators. Requests to access the datasets should be directed to the study principal investigator.

## Ethics Statement

This study was approved by the University of Illinois at Chicago Institutional Review Board (2015-0815 and 2016-0773) and the Chicago Department of Public Health (16-06). Written informed consent to participate in this study was provided by the participants' legal guardian/next of kin.

## Author Contributions

HL, MM, OP, NO, and GR made substantial contributions to conception and design of the work as well as analysis and interpretation of data and contributed to revision of this manuscript. NM-O'N and AS made substantial contributions to acquisition and analysis of data and revision of this manuscript. All authors approved publication of content and agree to be accountable for all aspects of the work in ensuring that questions related to the accuracy or integrity of any part of the work are appropriately investigated and resolved.

## Conflict of Interest

The authors declare that the research was conducted in the absence of any commercial or financial relationships that could be construed as a potential conflict of interest.

## Abbreviations

TBOS: Toothbrushing Behavior Observation System
CO-OP: Coordinated Oral health Promotion
CHW: community health worker
RA: research assistant
ICC: intraclass correlation coefficients.
