# Supplementary material for: Can an Instrument Validated to Assess Parent–Child Interactions in the Laboratory Setting Be Applied to Home-Based Observations?
Source: Front Pediatr. 2021 Jan 15;8:550922. doi: 10.3389/fped.2020.550922 (PMC7845142; doi:10.3389/fped.2020.550922)
Supplement: Supplementary file 1 [file Table_1.DOCX]

Supplementary Table 1. Comparison of TBOS Items vs. Feasible TBOS Items

|  | **TBOS** | **Feasible TBOS** |
| --- | --- | --- |
| **Parent Items n=5/12** | **n=12** | **n=5** |
| Offered one or more appropriate choices |  |  |
| Physically scaffolded the interaction |  |  |
| Prompted child to open his/her mouth wide |  |  |
| One or more positive messages to motivate |  |  |
| Laughed or smiled during toothbrushing |  |  |
| Used bribery to resolve problem behavior |  |  |
| 3 or more unlabeled praise statements related to toothbrushing |  |  |
| One or more negative remarks about or to the child |  |  |
| Used distraction to help child brush teeth |  |  |
| Yelled at child or raised voice |  |  |
| 1 or more labeled praise statements related to toothbrushing |  |  |
| Made threats |  |  |
| **Child Items** | **n=18** | **n=10** |
| Easily transitioned to toothbrushing |  |  |
| Imitated parent’s demonstrations of toothbrushing |  |  |
| Played with toothbrush, toothpaste, or water>50% of the time |  |  |
| Looked at parent’s face |  |  |
| Focused attention on toothbrushing >50% of the time |  |  |
| One or more verbal descriptions of toothbrushing |  |  |
| Sucked, bit, or chewed on toothbrush |  |  |
| Initiated gentle, social physical contact with parent |  |  |
| Gentle with parent and toothbrushing materials |  |  |
| Asked questions related to toothbrushing |  |  |
| “Bossy” towards parent |  |  |
| Responded to parent’s cues >50% of the time |  |  |
| Comforted by parent’s response to distress |  |  |
| Showed any distress |  |  |
| Showed any refusal behavior |  |  |
| Whined, complained, or other mild verbal protest |  |  |
| Yelled or screamed |  |  |
| One or more major refusal behaviors |  |  |

Parent and Child Items that were feasible to be coded using video-recordings based in urban home settings are compared to Parent and Child Items that comprise the original TBOS measure. All items that were feasible to code are indicated by shaded boxes.
